# Difficulty producing high-pitched sounds in singing: correlations with laryngostroboscopy and electromyographic findings^☆^

**DOI:** 10.1016/j.bjorl.2019.04.005

**Published:** 2019-05-18

**Authors:** Gustavo Polacow Korn, Renata Rangel Azevedo, Juliana Ceglio Monteiro, Denise Spinola Pinheiro, Sung Woo Park, Noemi Grigoletto de Biase

**Affiliations:** a Universidade Federal São Paulo, Departamento de Otorrinolaringologia e Cirurgia de Cabeça e Pescoço, São Paulo, SP, Brazil; b Universidade Federal São Paulo, Departamento de Fonoaudiologia, São Paulo, SP, Brazil; c Universidade Federal São Paulo, Departamento de Neurofisiologia, São Paulo, SP, Brazil

**Keywords:** Singing, Laryngoscopy, Stroboscopy, Electromyography, Canto, Laringoscopia, Estroboscopia, Eletromiografia

## Abstract

**Introduction:**

Difficulties or limitations in producing high-pitched sounds while singing may be due to the vocal technique used or organic factor. The observation of such difficulty or limitation by singing teachers is one of the main reasons affected individuals are referred to otolaryngologists.

**Objective:**

To evaluate the laryngostroboscopic and electromyographic changes in the cricothyroid muscles of singers with difficulties or limitations producing high-pitched sounds.

**Methods:**

This is a cross-sectional study. Ten singers with difficulty producing high-pitched sounds underwent voice, laryngostroboscopic, and electromyographic evaluations.

**Results:**

None of the evaluated singers presented signs of impairment of the superior laryngeal nerve on laryngostroboscopy. The electromyographic findings for the cricothyroid muscle were normal for all singers. Six singers presented vocal fold lesions, seven had signs suggestive of laryngopharyngeal reflux, and two presented vascular changes.

**Conclusion:**

No signs suggestive of superior laryngeal nerve paresis or paralysis were observed on laryngostroboscopy and electromyography of the cricothyroid muscle in singers with difficulties or limitations producing high-pitched sounds. The presence of vocal fold lesions should be investigated in this population.

## Resumo

**Introdução:**

Dificuldades ou limitações na produção de sons agudos durante o canto podem ser decorrentes da técnica vocal utilizada ou de fatores orgânicos. A observação dessa dificuldade ou limitação pelos professores de canto é uma das principais razões pelas quais os indivíduos afetados são encaminhados aos otorrinolaringologistas.

**Objetivo:**

Avaliar as alterações laringoestroboscópicas e eletromiográficas no músculo cricotireóideo de cantores com dificuldades ou limitações na produção de sons agudos.

**Método:**

Estudo de coorte transversal. Dez cantores com dificuldade de produzir sons agudos foram submetidos a avaliações de voz, laringoestroboscópicas e eletromiográficas.

**Resultados:**

Nenhum dos cantores avaliados apresentou sinais de comprometimento do nervo laríngeo superior na laringoestroboscopia. Os achados eletromiográficos para o músculo cricotireóideo foram normais para todos os cantores. Seis cantores apresentaram lesões nas pregas vocais, sete apresentaram sinais sugestivos de refluxo laringofaríngeo e dois apresentaram alterações vasculares.

**Conclusão:**

Não foram observados sinais sugestivos de paresia ou paralisia do nervo laríngeo superior na laringoestroboscopia e eletromiografia do músculo cricotireóideo em cantores com dificuldades ou limitações na produção de sons agudos. A presença de lesões nas pregas vocais deve ser investigada nessa população.

## Introduction

Difficulties or limitations in producing high-pitched sounds while singing may be due to the vocal technique; however, organic changes may be a contributing or causative factor.^1^ The observation of such difficulty or limitation by singing teachers is one of the main reasons the affected individuals are referred to otolaryngologists.

Previous studies have shown paresis of the superior laryngeal nerve (SLN) in singing students, which affected proper functioning of the cricothyroid (CT) muscle and resulted in difficulty singing.^1^, ^2^ SLN paresis or paralysis may lead to a decrease in pitch and impaired vocal performance, especially for high-pitched sounds.^1^ The diagnosis is “difficult”^1^ and “challenging”^3^, ^4^; consequently, this condition is often neglected^5^ or misdiagnosed as functional dysphonia.^6^ The resulting voice changes may be severe in voice professionals, particularly singers.^4^, ^6^

Impairment of the SLN/CT complex can be analyzed by voice assessment, laryngostroboscopic evaluation of laryngeal movement and electromyography.

In terms of voice assessment, auditory perceptual and aerodynamic parameters and the acoustic consequences of SLN injuries have not yet been accurately determined.^4^ Eckley et al.^2^ observed that vocal range and tessitura are useful parameters for analyzing the effects of SLN paralysis or paresis on the voice. In addition to limitations in producing high-pitched sounds, Dursun et al., in a study with preselected cohort of patients with suspected SLN injury (singers and non singers), found that this condition may lead to hoarseness, vocal fatigue, loss of volume, loss of projection, and breathiness.^1^ It is worth mentioning that most of these patients had a neuritis.^1^, ^2^ Roy et al.,^7^ in a simulation study using lidocaine to block the CT unilaterally in healthy males, found that patients with SLN injuries presented a decrease in phonatory frequency range with compression of both the upper and lowermost regions of the pitch range.

Some laryngoscopic signs suggest unilateral SLN paresis or paralysis, including rotation of the posterior part of the larynx to the impaired side, which leads to an oblique glottis,^5^, ^8^, ^9^, ^10^ shortening of the impaired vocal fold,^1^, ^10^ slower movement of the impaired vocal fold,^1^ deviation of the epiglottis petiole to the impaired side during the production of high-pitched sounds,^4^ arching of the impaired vocal fold,^10^, ^11^, ^12^ misalignment of the vocal folds during the production of high-pitched sounds (either the upper level^12^, ^13^ or the lower level^1^ of the fold may be affected, which may explain phase asymmetry),^13^ amplitude asymmetry (reduced on the affected side), and incomplete glottal closure.^14^ Moreover, anterior-posterior constriction and glottal hyperadduction may manifest to compensate for dysphonia secondary to muscle tension.^1^

However, the absence of signs such as shortened, thickened vocal folds with an oblique glottis and rotation of the larynx does not rule out SLN paralysis.^1^, ^11^, ^15^, ^16^, ^17^ Moreover, there is no consensus regarding which laryngeal finding should be considered pathognomonic.

Laryngeal electromyography (LEMG) is a well-tolerated, easily performed, minimally invasive examination that allows for the evaluation of laryngeal innervation, such as the SLN, as well as the CT muscle and other muscles.^18^ Furthermore, with this technique, the CT muscle is easily accessible.^9^ In cases of paresis, the examination may reveal polyphasic units with decreased recruitment and signs of partial denervation with regeneration. SLN paralysis may involve the absence of recruitment and the presence of fibrillation and positive waves.^1^, ^19^, ^20^ These are unequivocal signs of neuromuscular dysfunction, especially in cases in which the findings for the contralateral muscle are normal.^19^

No studies in the literature have correlated limitations or difficulty in producing high-pitched sounds in singing students as the sole symptom with changes in the innervation and function of the CT muscle.

Therefore, investigations of the electrophysiological activity of the CT muscle may contribute to the multidisciplinary assessment of singers with difficulty reaching high notes. The combination of electrophysiological and laryngostroboscopic findings allows assessment of the presence of lesions and signs suggestive of SLN paresis or paralysis. Some laryngoscopic findings as vocal fold edema, or lesion in the midmembranous vocal fold could justified this difficulty in singers.

Some questions could be raised. Is LEMG necessary for singers with difficulty singing high notes as the sole symptom? Would SLN compromising be common to find in singers with difficulty singing high notes as the sole symptom? In case of no signs of SLN compromising, by LEMG or laryngostroboscopic evaluation, what could be the explanation for this sole symptom? Is there lesions or alteration in the vocal fold midmembranous even without others symptoms? Or even inflammation caused by laryngopharyngeal reflux? Because of the possible effect of smoking in singing voice, smokers should not be part of the sample.

The objective of this study was to evaluate the possible causes of difficulty or limitations in producing high-pitched sounds in singers by laryngostroboscopic and electromyographic changes in the CT muscles.

## Methods

This prospective study was approved by the Research Ethics Committee of the Federal University of São Paulo under Opinion no. 1.156.506.

The sample consisted of ten singers (selected by their singing teachers) who exhibited difficulty or limitations in producing high-pitched sounds and were recruited between July 2015 and July 2016. All participants were required to sign an informed consent form approved by the institutional Research Ethics Committee prior to inclusion. These participants represent a pilot sample because no data on the prevalence of the evaluated changes are available in the literature.

Difficulty or limitations in producing high-pitched sounds was evaluated only by each singer's teacher and not by the singer him/herself. Each singer teacher observed difficulty or limitations in producing high-pitched sounds even after working with singing technique.

Singers of both sexes who met the following criteria were included: (1) participation in singing class for at least three months; (2) difficulty producing high-pitched sounds as the only complaint and (3) age of 18–60 years.

The exclusion criteria were (1) a history of hoarseness in the past 12 months; (2) a history of previous treatment for hoarseness; (3) chronic diseases, such as thyroid disease and diabetes mellitus; (4) use of psychotropic medications; (5) smoking; (6) pulmonary diseases; (7) use of anticoagulants or a diagnosis of coagulopathy and (8) age <18 or >60 years because of the possible effects of presbyphonia and voice mutation.

The study sample consisted of six women and four men. The mean age was 32 ± 8 years among the women and 35 ± 17.5 years among the men, without a significant difference between the two groups (*p* = 0.831).

The singers were assessed in three phases – voice assessment, laryngostroboscopy, and laryngeal electromyography – and on different dates. Each participant's voice was assessed by two speech-language pathologists with more than ten years of experience in voice disorders. The laryngostroboscopy results were evaluated by two examiners with more than 10 years of practice. In cases of disagreement, the video of the examination was repeated and discussed until a consensus was reached. Electromyography was conducted by an otorhinolaryngologist and a neurophysiologist, that latter of whom was blind to the laryngostroboscopic findings.

During the voice assessments, the singers’ voices were recorded during the production of vowel sounds. The voice samples were analyzed acoustically using the Voice Laboratory Program (Praat)^21^ considering the following acoustic parameters: fundamental frequency (*F*_0_), jitter and shimmer, the harmonics-to-noise ratio (HNR), minimum and maximum voice frequencies, and the frequency range in semitones. Voice assessment was performed solely to describe our sample.

Laryngoscopy was performed with a voice laryngoscope (Wolf 70°) and a nasofibrolaryngoscope (Pentax) connected to a light source and video. Stroboscopy was conducted using a standard system (Ecleris, Argentina). The patient remained seated during the examination, and during videolaryngoscopy, the tongue was protruded outwards and held in that position by the examiner using gauze. The following tasks were performed: production of a comfortable /e/, a high-pitched /i/ and alternate production of “i” and “sniff.” The following parameters were evaluated on laryngostroboscopy: (1) the presence of minimal structural lesions in the vocal fold mucosa; (2) the presence of vocal fold lesions; (3) changes in the mobility of the vocal folds during the alternate production of “i” and “sniff;” (4) rotation of the larynx with an oblique glottis during the production of high-pitched sounds; (5) suspected vocal fold height mismatch; (6) deviation of the epiglottis petiole during the production of high-pitched sounds; (7) the presence of phase or amplitude asymmetry and glottal closure on stroboscopy and (8) the presence of signs suggestive of laryngopharyngeal reflux, including interarytenoid hyperemia, retrocricoid edema, and pseudo-sulcus.

On electromyography, only the CT muscle, which is responsible for increasing the voice frequency, was investigated. A Nihon Kohden Neuropack 1 device was used. The singer's neck remained extended, and the skin was cleaned with 70% alcohol. After the CT membrane was identified, the needle of the concentric electrode (Spes Medica) was inserted approximately 0.5 cm from the midline and directed laterally at an angle of 30° to 45°. The needle crossed the sternohyoid muscle and reached the CT muscle, which is located at a depth of approximately 1 cm. To confirm the position of the electrode, the patient was asked to produce a low-pitched /i/ and then a high-pitched /i/ and a marked increase in the EMG signal was expected.^22^ Topical anesthesia was not used because it can lead to changes in the electrical signal and confound the results.^23^, ^24^ The evaluated neurophysiological parameters included insertional activity during electrode positioning, muscle activity at rest, and minimal and maximal muscle contractions during the production of a weak high-pitched /i/ followed by a strong high-pitched /i/.

The insertional activity observed during insertion of the electrode into the muscle corresponded to mechanical depolarization of the muscle fiber. In healthy muscles, insertional activity produces small electrical potentials that persist for short periods after interrupting electrode movement. However, these potentials may be increased or decreased by pathological processes. Muscle activity at rest refers to muscle activity without voluntary control. This type of activity is not observed in healthy muscles, and when present (in the form of fibrillation and positive sharp waves), it may be due to myopathic or neurogenic disorders. Minimal muscle contraction is used to evaluate the action potential of motor units. The recruitment pattern of motor units, i.e., how motor units are activated by a progressive increase in force,^25^ is analyzed during moderate or maximal muscle contraction.

The data were subjected to descriptive analysis.

## Results

The results of the acoustic parameter analysis are described in Table 1.

**Table 1:** Analysis of the acoustic parameters.

Gender	*F*_0_ (Hz)	Jitter (%)	Shimmer (%)	HNR	Min (Hz)	Max (Hz)	*F*_0_ range (st)
Women	182	0.218	2.148	0.004	142	567	2
	209	0.243	2.671	0.005	156	625	2
	219	0.185	2.140	0.005	148	555	2
	197	0.268	2.165	0.005	153	532	2
	252	0.237	1.944	0.003	156	734	3
	196	0.166	2.763	0.004	144	854	1
Men	149	0.412	3.914	0.022	85	455	1
	152	0.335	2.782	0.004	98	380	1
	129	0.172	3.062	0.006	72	422	1
	129	0.180	3.212	0.006	77	370	2

*F*_0_ (in Hz), fundamental frequency; HNR, harmonics-to-noise ratio, Min (in Hz), minimum frequency; Max (in Hz), maximum frequency; *F*_0_ range (st), frequency range in semitones.

The laryngostroboscopic results are shown in Table 2. None of the evaluated singers showed signs of impaired vocal fold mobility, rotation of the larynx with an oblique glottis during the production of high-pitched sounds, vocal fold height mismatch, or deviation of the epiglottis petiole during the production of high-pitched sounds. Six of the ten patients presented vocal fold lesions, seven had signs suggestive of laryngopharyngeal reflux, and two exhibited vascular changes. The stroboscopic findings indicated that three patients presented phase asymmetry, one had amplitude asymmetry, and four exhibited incomplete glottal closure, including two with a medium-posterior triangular chink and two with an anterior fusiform chink.

**Table 2:** Laryngostroboscopic findings.

Variables	*n* (%)
*Presence of vascular changes*	2 (20)

*Vocal fold lesions*	6 (60)
Small polyp in the anterior to middle third of the right vocal fold	1
Bilateral thickening of the middle third of the vocal folds	2
Edema in the middle third of the left vocal fold	1
Small of vocal fold nodules	1
Bilateral thickening between the anterior and middle third of the vocal folds	1

*Signs of laryngopharyngeal reflux*	7 (70)
Interarytenoid hyperemia	2
Interarytenoid hyperemia and retrocricoid edema	3
Pseudo-sulcus, interarytenoid hyperemia, and retrocricoid edema	2

*Signs of SLN compromising*	
Alternate production of “i” and “sniff”	0 (0)
Rotation of the larynx	0 (0)
Vocal fold height mismatch	0 (0)
Deviation of the epiglottis petiole	0 (0)

*Stroboscopic findings*	
Periodicity	9 (90)
Phase asymmetry	3 (33.3)
Amplitude asymmetry	1 (11.1)

*Glottal closure*	
Preserved glottal closure	6 (60)
Anterior fusiform chink	2 (20)
Medium-posterior triangular chink	2 (20)

Electromyography showed no significant changes of the CT muscles of the evaluated singers (Table 3).

**Table 3:** Results of laryngeal electromyography of the cricothyroid muscle according to gender.

Gender	Insertional activity during electrode positioning	Muscle activity at rest	Minimal muscle contraction	Maximal muscle contraction
Women	Normal	Normal	Normal	Normal
	Normal	Normal	Normal	Normal
	Normal	Normal	Normal	Normal
	Normal	Normal	Normal	Normal
	Normal	Normal	Normal	Normal
	Normal	Normal	Normal	Normal

Men	Normal	Normal	Normal	Normal
	Normal	Normal	Normal	Normal
	Normal	Normal	Normal	Normal
	Normal	Normal	Normal	Normal

## Discussion

Although the singers in the sample were aware of their difficulty producing high-pitched sounds, they did not seek medical care, suggesting that other singers with similar complaints do not seek medical care. One reason for not seeking medical care may be financial or insurance coverage limitations.^26^

One of the hypotheses of this study was that singers whose only vocal complaint is difficulty producing high-pitched sounds could present signs of SLN impairment on laryngostroboscopy, electromyography, or both. However, the laryngostroboscopic and electromyographic findings indicated that none of the evaluated patients had signs of SLN impairment.

As there is no consensus regarding which laryngeal finding should be considered pathognomonic of SLN/CT impairment, the analysis of the laryngostroboscopic evaluation plus LEMG is of fundamental importance for the evaluation of the SLN/CT complex.

Some studies have diagnosed patients with SLN paresis and paralysis using laryngostroboscopy and laryngeal electromyography. However, in these studies, the complaints that led patients to seek medical care were not restricted to difficulty producing high-pitched sounds. Dursun et al.^1^ reported that the main complaints among 126 patients with SLN paresis or paralysis were vocal fatigue (82.5%), hoarseness (75.4%), volume disturbance (75.4%), loss of modulation (69%), and breathiness (34.9%). Eckley et al.^2^ evaluated 30 singers with the same dysfunction and found that their main symptoms were hoarseness (53.6%), loss of ability to produce high-pitched sounds (46.2%), vocal fatigue (39.3%), breathiness (30.3%) and volume disturbance (25%).

Because difficulty producing high-pitched sounds was the only complaint among singers who did not seek medical care, we hypothesized that paresis was less likely. However, other organic changes, such as vocal fold lesions, may be involved.

In this study, voice assessment was performed solely to describe our sample. Unfortunately, we did not find voice evaluation data for singers with difficulty producing high-pitched sounds in the literature to compare with our findings.

Regarding laryngostroboscopic findings, the presence of vocal fold lesions in 60% of our sample could justify difficulty in producing high-pitched sounds. This percentage was higher than that reported in other studies involving healthy singers (6%)^27^ and asymptomatic singing students (in terms of vocal fold lesions and edema; 38.6%)^28^ but similar to the percentage found in young singers (78.4%) at enrollment in an elite opera conservatory.^29^

The percentage of patients with vascular changes in our sample (20%) was similar to that of healthy singing teachers (18.1%),^30^ higher than that of healthy singers (3.0%)^27^ and lower than that of young singers (31.4%) assessed at enrollment in an elite opera conservatory.^29^

The rate of incomplete glottal closure (40%) is similar to that reported for healthy singing teachers (34.7%)^30^ and young singers (46%) at enrollment in an elite opera conservatory^29^ but lower than that of asymptomatic singing students (84.1%).^28^ The rate of phase asymmetry in our sample was 30%, which is similar to that of young singers at enrollment in an elite opera conservatory (26%)^29^ but higher than that of healthy singing teachers (9.7%).^30^ The rate of amplitude asymmetry was 10%, which is similar to that found in healthy singing teachers (5.6%)^30^ but lower than that of asymptomatic singing students (38.6%)^28^ and young singers at enrollment in an elite opera conservatory (36%).^29^ Unfortunately, we were unable to find other studies that evaluated singers whose only complaint was difficulty producing high-pitched sounds, which hindered the comparison results.

The rate of laryngopharyngeal reflux signs was high in our sample (70%). This finding is not uncommon; signs of laryngopharyngeal reflux have been reported in 42% of healthy singers,^27^ 72% of healthy singing teachers,^30^ 73.4% of asymptomatic singing students^28^ and 69% of young singers at enrollment in an elite opera conservatory.^29^ This type of reflux seems to be common in singers, and stress, behavioral patterns related to performance demands, and increased intra-abdominal pressure may contribute to this complication. The comparison of young singers assessed at enrollment in an elite opera conservatory^29^ and patients at follow-up indicated that the only significant change was an increase in laryngopharyngeal reflux signs.

Unilateral or bilateral impairment of vocal fold mobility was absent in our sample but was observed in 1.5% of healthy singers,^27^ 15.3% of healthy singing teachers^30^ and 37.7% of young singers at enrollment in an elite opera conservatory.^29^ Notably, in these studies, suspected changes in mobility and paresis were evaluated using laryngostroboscopy alone, without laryngeal electromyography.

Some vocal fold lesion could be accompanied by vocal fold paresis. Even though a paralysis or paresis is an uncommon diagnosis, LEMG evaluation was important to rule out signs of SLN compromising.

Smoker singers were not part of our sample because of the influence of the fundamental frequency on speaking voice.^31^

A limitation of our study was sample recruitment, as the exclusion of singers who were recently treated or undergoing treatment and singers with other vocal complaints reduced the potential sample size. Another limitation was non-adherence of the volunteers, which may have occurred for social reasons. Besides that, our sample was constituted by singers referred by his or her singer teacher that observed difficulty in reaching high-notes, and not by a specific evaluation of the singing technique.

Currently, little is known regarding the epidemiology of vocal disorders, particularly in voice professionals (Phyland et al., 1999).^32^ Further studies are necessary to better understand singers as a population and to offer better guidelines regarding the need for medical care.

Based on our findings, we suggest that all singers with difficulty producing high-pitched sounds should undergo medical examinations for the early diagnosis and timely treatment of the cause, especially in cases of vocal fold lesions and laryngopharyngeal reflux.

## Conclusion

No signs suggestive of SLN paresis or paralysis were found on laryngostroboscopy and electromyography of the CT muscle in singers with difficulty producing high-pitched sounds. Vocal fold lesions and laryngopharyngeal reflux were the most common findings in this population.

## Conflicts of interest

The authors declare no conflicts of interest.
